# Deciphering the FGFR2 Code: Innovative Targets in Gastric Cancer Therapy

**DOI:** 10.3390/curroncol31080321

**Published:** 2024-07-29

**Authors:** Alireza Tojjari, Sarbajeet Nagdas, Ali Saeed, Anwaar Saeed

**Affiliations:** 1 Department of Medicine, Division of Hematology & Oncology, University of Pittsburgh Medical Center (UPMC), Pittsburgh, PA 15261, USA; alirezatojjari@gmail.com; 2 UPMC Hillman Cancer Center, Pittsburgh, PA 15232, USA; nagdass@upmc.edu; 3 Department of Medicine, Ochsner Lafayette General Medical Center, Lafayette, LA 70503, USA; asaeedmd@gmail.com

**Keywords:** FGFR2, FGFR2-targeted therapy, gastric cancer biomarkers, molecular oncology, precision medicine

## Abstract

Gastric cancer (GC) represents a major global health challenge as a highly prevalent disease with high mortality whose global incidence and mortality are predicted to worsen over the coming years. To date, our standard of care for advanced gastric cancer of combination chemotherapy and immunotherapy has a 1-year overall survival rate of 55%. Significant efforts have gone into identifying targetable alterations in gastric cancer, ultimately yielding the Fibroblast Growth Factor Receptors (FGFRs) family, specifically FGFR2 as a promising target. FGFR2 is overexpressed in GC, particularly diffuse-type GC, and is associated with poor prognostic outcomes. In recent years, there has been an increasing number of small molecule inhibitors and monoclonal antibodies targeting FGFR2 that have entered into clinical trials. Specifically for GC, these agents are currently being trialed in various phases as monotherapies or with standard-of-care treatments to make a clinically meaningful impact on what appears to be an important biological axis of GC. In this review, we outline the underlying biology of FGFR2, its putative role in GC, and the various FGFR2-targeted agents currently in clinical trials for gastric cancer patients as well as postulate some challenges in adopting these therapeutics for clinically meaningful benefit.

## 1. Introduction

Gastric cancer (GC) represents a major global health challenge, ranking as the fifth most prevalent cancer and the fourth leading cause of cancer-related mortality worldwide. This malignancy not only impacts a significant number of individuals annually but also poses a growing public health concern due to increasing risk factors such as obesity and Helicobacter pylori infection, particularly in Asian countries. The projected rise in both incidence and mortality underscores the critical need for enhanced diagnostic and therapeutic approaches [1].

For a long time, the backbone of therapy for advanced GC has been the use of fluoropyrimidine and platinum-based chemotherapeutics. The advent of advanced molecular and genomic studies into GC has allowed for better characterization of this disease. The Cancer Genome Atlas (TCGA) genomic analysis identified four distinct subtypes in gastric cancer: tumors characterized by microsatellite instability (MSI), those positive for the Epstein–Barr virus (EBV), tumors with chromosomal instability (CIN), and those identified as genomically stable (GS) tumors [2]. Despite these approaches, the search for molecular markers that can predict responses to treatments or are targetable with clinically meaningful benefits has not been as promising as previously hoped. For instance, early efforts were focused on targeting Receptor Tyrosine Kinase (RTK) pathway proteins such as Epidermal Growth Factor Receptor (EGFR), Hepatocyte Growth Factor Receptor (MET), and Vascular Endothelial Growth Factor Receptor (VEGFR), achieving limited success. Fortunately, after 2010, more efficacious targeted therapy was demonstrated, best exemplified by the adoption of trastuzumab for Human Epidermal Growth Factor Receptor 2 (HER-2)-positive gastric cancer. The success of trastuzumab marked a considerable step forward in integrating targeted therapy with traditional chemotherapy based on advanced molecular diagnostic testing and serves as an impetus to find additional options for patients [3,4,5,6]. 

The Fibroblast Growth Factor Receptors (FGFRs) are a family of RTKs that play a crucial role in physiologic and oncogenic signaling through interactions with fibroblast growth factors (FGFs) [7]. Recent research has elucidated the evolving significance of FGFs and FGFRs in the development of certain subtypes of gastric cancer, attributed to their distinct molecular properties [8]. The FGFR signaling axis has become a promising avenue for developing new cancer treatments. FGFR signaling plays a crucial role in regulating various cellular functions such as growth, survival, and differentiation via different effector pathways such as the Mitogen-Activated Protein Kinase (MAPK) cascade [9]. Disruption in FGFR signaling is linked to the onset of tumorigenesis and cancer progression. Specifically, amplification of the FGFR2 gene stands out as the predominant anomaly within the FGFR2 gene, particularly influencing GCaGC and especially its diffuse form, which comprises more than one-third of all GC cases [2,10,11,12]. FGFR2 overexpression via immunohistochemistry (IHC) is observed in 31–61% of gastric cancer cases and correlates with aggressive tumor characteristics such as advanced T stage, increased lymph node metastasis, and reduced overall survival rates, highlighting its potential as a therapeutic target [13,14,15]. FGFR2 mutations and fusions are not exclusive to gastric cancer. These genetic alterations have been identified in several other cancers, including bile duct cancer (cholangiocarcinoma), breast cancer, lung cancer, and endometrial cancer. In cholangiocarcinoma, FGFR2 fusions are present in approximately 10–15% of cases and are associated with younger patient age and a unique histological subtype [13,14]. In breast cancer, FGFR2 amplifications are found in about 5–10% of cases, particularly in hormone-receptor-positive subtypes [15]. Lung squamous cell carcinoma and endometrial cancer also exhibit FGFR2 alterations, though less frequently [16]. These mutations and fusions contribute to oncogenesis by promoting cell proliferation, survival, and migration, thereby playing a crucial role in the pathophysiology of these cancers [9].

The biological impact of FGFR2 mutations and fusions in these cancers is considerable. These alterations cause abnormal FGFR2 signaling, which in turn triggers downstream pathways like MAPK, PI3K/AKT, and STAT, leading to increased tumor proliferation and survival [9]. Grasping these effects is essential for creating targeted treatments and enhancing patient prognosis.

The importance of FGFR2 in gastric cancer is underscored by its genetic alterations, role in oncogenic signaling pathways, and potential as a therapeutic target. FGFR2 amplifications and mutations are common in gastric cancer, especially in the diffuse subtype, with overexpression noted in 31–61% of cases. These genetic changes are linked to poor prognosis, including advanced T stage, increased lymph node metastasis, and lower overall survival rates [17,18]. Disruptions in FGFR2 signaling contribute to tumor development and cancer progression. FGFR2 is recognized as a valuable therapeutic target in gastric cancer. The creation of small molecule inhibitors and monoclonal antibodies targeting FGFR2 has shown promise in clinical trials, emphasizing the potential of FGFR2 in targeted therapies for gastric cancer [9,13,19].

In the following sections, we will delve deeper into the role of FGFR2 in GC, exploring its biological functions, its contribution to cancer pathogenesis, and the potential benefits and challenges of targeting FGFR2 as a therapeutic strategy. The literature included in this review encompasses peer-reviewed research articles, clinical trials, and reviews that specifically investigate FGFR2 in gastric cancer. Non-peer-reviewed sources and studies not addressing FGFR2 in GC were excluded. Through this comprehensive analysis, we aim to highlight the significance of FGFR2 as an emerging target in the battle against GC, paving the way for more personalized and effective treatment approaches.

## 2. Background

### 2.1. First Look at FGFs and FGFRs

#### 2.1.1. A Primer to FGFs

Fibroblast Growth Factors (FGFs) are a family of proteins involved in various biological processes. Initially discovered in the 1970s as mitogens extracted from bovine brain tissue, FGFs have been recognized for their diverse biological functions. These factors promote cellular proliferation, survival, migration, and differentiation and serve as powerful agents in angiogenesis and wound repair [20]. The canonical FGFs are grouped into specific subfamilies based on their mode of action, either paracrine or autocrine, including FGF1 (FGF1/2), FGF4 (FGF4/5/6), FGF7 (FGF3/7/10/22), FGF8 (FGF8/17/18), and FGF9 (FGF9/16/20). These subfamilies function by binding to and activating FGFRs. The presence of heparan sulfate proteoglycan (HSPG) co-receptors facilitates the activation process, which stabilizes the interaction. This stabilization is critical for the formation of the FGF/FGFR/HSPG ternary complex, which in turn activates various signaling cascades and thus promotes FGF/FGFR physiological activities [21]. The FGF15/19 subfamily, including FGF15/19/21/23, represents a unique cluster of endocrine growth factors within the larger FGF family. These factors distinguish themselves through their necessity for α- and β-Klotho proteins as co-receptors and play crucial roles in managing various metabolic functions. In contrast, the FGF11 subfamily, consisting of FGF11/12/13/14, operates through an ‘intracrine’ or intracellular mechanism, pivotal in regulating neuronal and myocardial functions [22]. 

As such, FGFs and FGFRs play a pivotal role in the development and homeostatic functionality of various organ systems. The mammalian family of FGFs has 22 known members, with most being secreted glycoproteins. Among the FGF family, FGF-1 (also known as acidic FGF) and FGF-2 (basic FGF) are distinguished by their export from the cell through not yet fully elucidated mechanisms, in contrast to other FGFs that utilize the conventional secretory pathway. Due to their strong affinity for the glycosaminoglycan side chains on cell surface proteoglycans, these secreted FGFs are believed to be retained either on the surface of the cell that secretes them or on adjacent cells, facilitating their role in autocrine or paracrine signaling.

#### 2.1.2. A Closer Look at FGFRs

Fibroblast Growth Factor Receptors (FGFRs) are transmembrane tyrosine kinases and part of the immunoglobulin (Ig) superfamily. The FGFR family consists of four members known as RTKs: FGFR1, FGFR2, FGFR3, and FGFR4. Additionally, there is a fifth receptor, FGFRL1, which, despite being related, does not contain a tyrosine kinase domain and may play a role in negatively regulating signaling [9]. FGFRs are characterized by a uniform structure encompassing three primary components: an extensive extracellular domain for ligand binding, a singular transmembrane helix, and a cytoplasmic tyrosine kinase domain. The extracellular domain houses the site for FGF binding, featuring up to three Ig-like loops generated through alternative splicing processes. The loops closest to the membrane are responsible for binding to the FGF ligands. This interaction leads to the creation of a complex that comprises a minimum of two FGF molecules, two FGFRs, and a glycosaminoglycan component, facilitating the specific cellular responses triggered by FGFs [23,24,25]. This receptor dimerization and complex formation induces structural alterations in the intracellular domains that activate and phosphorylate the tyrosine kinase domain. Activated FGFRs in turn engage with and activate various effector pathways, like the MAPK pathway. 

**Figure 1:**
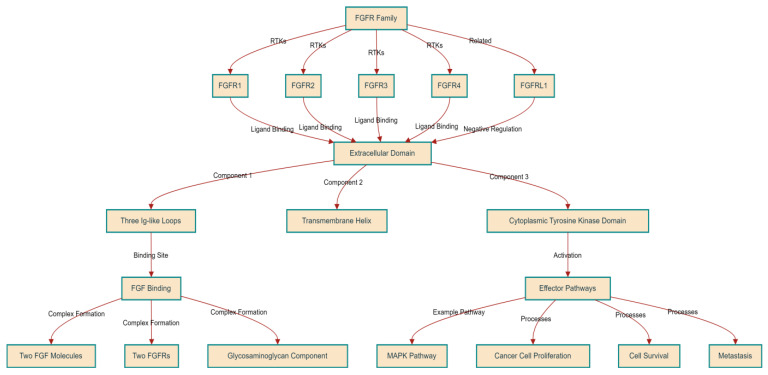
Structural and Functional Overview of the FGFR Family.

FGFR signaling intricately interacts with downstream pathways critical for cancer cell proliferation, survival, and metastasis [24]. Figure 1 summarizes the structure and function of the FGFR family.

### 2.2. FGFR Signaling in Cancer Progression

Similar to many other RTKs, FGFR-mediated signaling is vital for the normal development and homeostatic maintenance of bodily tissues. However, aberrations in FGFR signaling, particularly those that increase signaling, are increasingly recognized as key drivers in the initiation and progression of various cancers. 

#### 2.2.1. Genetic and Post-Transcriptional Alterations of FGFR

Genomic level changes in the FGFR loci have been found in different tumor types that promote oncogenesis. For instance, FGFR1 amplification has been implicated in the pathogenesis of breast cancer and squamous cell lung cancer [9] The amplification of FGFR genes leads to FGFR protein overexpression and enhanced oncogenic signaling. The increased gene dosage fosters an environment conducive to tumor growth even with relatively unchanged amounts of ligand. Chromosomal rearrangements involving FGFR genes can generate fusion proteins with aberrant regulatory and signaling capabilities [15]. Point mutations within the FGFR genes can constitutively activate receptors independent of FGF ligand binding, and promote oncogenic physiology such as cell proliferation and survival independent. For example, mutations in FGFR3 are linked to the development of bladder cancer and multiple myeloma [25]. Alternative splicing produces FGFR isoforms with distinct ligand-binding and signaling properties, contributing to cancer heterogeneity and progression. This is best highlighted with the switch from the FGFR3-IIIb isoform to the oncogenic FGFR3-IIIc isoform; this alternative splicing event has been shown to promote invasiveness in prostate and bladder cancers [26,27]. Genomic alterations, such as FGFR2 amplification, lead to protein overexpression, enhancing oncogenic signaling and promoting tumor growth even with low ligand levels [28]. Chromosomal rearrangements, like FGFR3–TACC3 fusion identified in glioblastoma, exemplify how such genetic alterations can promote malignant transformation [9]. Post-transcriptional modifications, such as alternative splicing, generate isoforms with distinct ligand-binding and signaling properties, contributing to cancer heterogeneity and progression [27].

#### 2.2.2. Effector Pathway Activation and Physiologic Impact

FGFR signaling interacts with downstream pathways critical for cancer cell proliferation, survival, and metastasis. Like other RTKs, activated FGFR can activate well-characterized oncogenic signaling networks like the phosphoinositide 3-kinase/protein kinase B (PI3K/AKT), MAPK, and Signal Transducer and Activator of Transcription 3 (STAT3) pathways, amongst many other signaling cascades [29]. Activation of these pathways has previously exhaustively been reviewed and shown to promote cell proliferation, cellular growth and survival, metabolic changes, and further transcriptional reprogramming [30]. Furthermore, FGFRs have been well characterized in their role in promoting angiogenesis, thereby promoting a pro-tumorigenic microenvironment. FGFRs also modulate the tumor microenvironment by influencing cancer cell interactions with stromal cells and the extracellular matrix, facilitating cellular migration and invasion [31]. 

### 2.3. FGFR2 in Gastric Malignancy

The abnormal activation of FGFR2 through mutations, gene amplification, or overexpression is a significant factor in the development of several types of cancer, including gastric cancer. FGFR2 overexpression is closely associated with poor survival outcomes and advanced disease stages, highlighting its critical role in gastric carcinogenesis. FGFR2 gene amplification acts as an adverse prognostic marker, closely linked with lymph node metastasis, poorly differentiated adenocarcinoma, and reduced survival [32]. Recent investigations have identified FGFR2 amplification as a significant aberration, particularly in patients with diffuse-type gastric cancer; this genetic alteration correlates with lower survival rates and a worse prognosis [33,34,35]. This dysregulation aids cancer’s progression by promoting unchecked cellular growth, evasion of programmed cell death, and increased tumor blood vessel formation [9].

The FGFR2 gene, located on the 10q26 region of human chromosome 10, is responsible for producing two splice variants, FGFR2b and FGFR2c. Despite their similarities, including the presence of extracellular immunoglobulin-like domains and a cytoplasmic tyrosine kinase domain, FGFR2b and FGFR2c differ in the latter part of their third immunoglobulin-like domain [36,37]. Specifically, FGFR2b is expressed on epithelial cells and has a high affinity for FGF1, FGF3, FGF7, FGF10, and FGF22. Conversely, FGFR2c is found in mesenchymal cells, where it shows a high affinity for FGF1, FGF2, FGF4, FGF6, FGF9, FGF16, and FGF20. This demonstrates the unique expression patterns and ligand specificity between FGFR2b and FGFR2c, underlining their specialized roles in cell signaling and interaction [38,39].

The FGFR2 splice variants act as receptors for FGFs, playing a crucial role in transmitting signals for the FGF to various effector pathways, some of which we highlight. FGFR2 phosphorylation activates the MAPK and PI3K/AKT pathways via Fibroblast growth factor receptor substrate 2 (FRS2). FGFR2 has also been shown to activate PI3K/AKT-mediated signaling via thrombospondin-1 (THBS1). Additionally, the FGFR2 variants engage in signaling through the Diacylglycerol/Protein Kinase C (DAG/PKC) and Inositol Trisphosphate/Calmodulin pathways. Furthermore, FGFR2 signaling activates the Yes-Associated Protein 1 (YAP1) pathway, most prominently demonstrated via FGF18–FGFR2 interactions [40]. Various studies have shown that activation of these various effector pathways promotes many hallmarks of cancer like cell cycle progression, cell growth inhibiting apoptosis, cell migration and invasion, and angiogenesis. 

Initial research into inhibitors that block FGFR2 or its related pathways has shown promising results, offering a potential avenue to improve outcomes for patients with FGFR2-related gastric cancer [41]. However, implementing FGFR2-focused therapies in clinical settings presents challenges, such as developing resistance and identifying patients who are most likely to benefit from these treatments. The diversity of gastric cancer cases requires detailed genomic profiling to customize treatment plans based on each patient’s cancer’s molecular characteristics. Ongoing efforts aim to understand resistance mechanisms to FGFR2 inhibitors and develop combination therapies to overcome these barriers [42].

FGFR2 overexpression is closely associated with poor survival outcomes and advanced disease stages. FGFR2 gene amplification acts as an adverse prognostic marker linked with lymph node metastasis and poorly differentiated adenocarcinoma [32]. This dysregulation promotes cancer progression by enhancing cellular growth, evasion of apoptosis, and increased tumor blood vessel formation [10,11].

## 3. Therapeutic Strategies Targeting FGFR2 in Gastric Cancer in Clinical Trials

FGFR2’s dysregulated activity, whether through amplification, mutations, or heightened expression, is closely linked to the development and progression of gastric cancer. This insight positions FGFR2 as a key candidate for therapeutic targeting. This narrative synthesizes the latest advances and clinical progress in therapies aimed at FGFR2, reflecting the cutting-edge research in this domain [43].

### 3.1. FGFR Inhibitors

**Figure 2:**
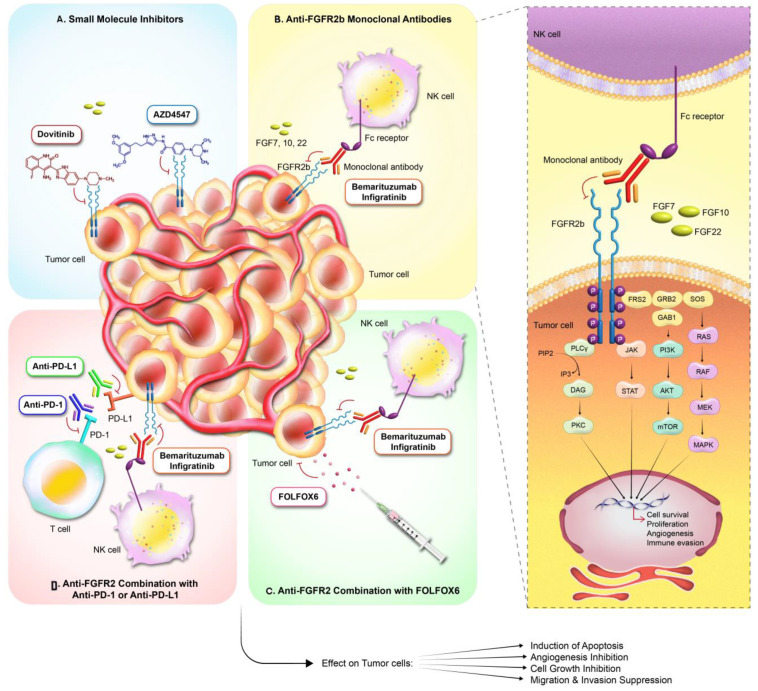
Targeted Therapies in FGFR2-Driven Cancers: This figure summarizes the multi-modal strategies for targeting the FGFR2 signaling pathway in oncology. (**A**) Small molecule inhibitors (e.g., dovitinib, AZD4547) penetrate tumor cells to directly inhibit the FGFR2 kinase activity. (**B**) Anti-FGFR2b monoclonal antibodies (e.g., bemarituzumab, infritinib) bind to FGFR2 on tumor cells, with potential for receptor inhibition and natural killer (NK)-cell-mediated cytotoxicity via Fc receptor engagement. (**C**) Anti-FGFR2 therapy combined with the chemotherapy regimen FOLFOX6 may augment cancer cell apoptosis, inhibit angiogenesis, and suppress cell growth, migration, and invasion. (**D**) Combination therapies utilizing anti-FGFR2 agents with immune checkpoint inhibitors (anti-PD-1, anti-PD-L1) aim to potentiate T-cell-mediated responses against the tumor. The intricate network of intracellular signaling stimulated by FGFR2 activation and its downstream oncogenic processes are depicted on the right, illustrating the intervention points for the various therapeutic agents and their impact on tumor cell fate.

The therapeutic landscape for gastric cancer includes small molecule inhibitors that act on FGFR2’s tyrosine kinase domain. These compounds interfere with the kinase function of FGFR2, disrupting the cell signals essential for the cancer cells’ growth and persistence. Among these, dovitinib and AZD4547 stand out in their efficacy in FGFR2-amplified gastric cancer models (Figure 2A). Clinically, initial phase II studies of dovotinib monotherapy in patients with advanced malignancy progressing on standard-of-care treatments (not specifically gastric cancer) revealed a median progression-free survival (PFS) of 2.4 months [44]. Indeed, this response in advanced solid malignancy, as well as preclinical data in FGFR2-amplified gastric cancers to dovitinib, sparked the GASDOVI phase II clinical trial investigating dovitinib in FGFR2-amplified gastric cancer; the result of this has yet to be publicly reported.

Such targeted approaches emphasize the value of detailed molecular characterization in customizing treatment plans for gastric cancer and underscore the need for a nuanced understanding of advanced malignancies’ complexity. Resistance to FGFR inhibitors in gastric cancer can arise through multiple mechanisms, including secondary mutations in the FGFR2 gene that prevent inhibitor binding, activation of compensatory signaling pathways such as MET and HER2, and changes in the tumor microenvironment that promote resistance. Understanding these resistance mechanisms is crucial for developing combination therapies to overcome resistance and enhance treatment efficacy [25,42].

To that end, many different approaches targeting FGFR2 in gastric cancer have progressed to the clinical space to date, which we summarize in Table 1. 

### 3.2. Anti-FGFR2b Monoclonal Antibodies

While many correlative studies and new treatment avenues have highlighted FGFR2 as a potential target for gastric cancer, preclinical models have highlighted FGFR2b as a particularly attractive target due to its pro-tumorigenic functions and specificity. Unfortunately, the previously highlighted targeted approaches inadequately target FGFR2 isoforms with any great specificity. However, the use of monoclonal antibodies engineered to zero in on FGFR2b leads the charge in innovative treatments. 

Among the various FGFR2b-directed monoclonal antibodies, bemarituzumab stands out as an agent that has progressed significantly clinically. Bemarituzumab binds to the ligand-binding domain on FGFR2b, inhibiting the FGF:FGFR2b activating interactions, thereby curtailing the activation of FGFR2b and its downstream signaling pathways (Figure 2B). The initial signals of clinical benefit were demonstrated in the FIGHT trial [45], investigating bemarituzumab with chemotherapy in patients with FGFR2b-selected gastric cancers. While this trial did not reach any statistically significant endpoint, the clinical benefit seen in the bemarituzumab arm with a PFS of 9.5 months sparked excitement for this agent. Currently, bemarituzumab’s effectiveness is being scrutinized in the follow-up phase III clinical trial, FORTITUDE, for patients diagnosed with FGFR2b-positive, advanced gastric cancer. Unlike FIGHT, FORTITUDE is designed to assess bemarituzumab with chemotherapy in previously untreated advanced gastric or gastroesophageal junction cancer with FGFR2b overexpression (Figure 2C) [46]. The study aims to evaluate the efficacy of bemarituzumab as part of first-line treatment, reflecting the ongoing effort to integrate FGFR2b-targeted therapy into the treatment paradigm for gastric cancer. This trial represents a crucial stride toward the incorporation of FGFR2b-targeted therapies into the realm of standard clinical care [35]. Some of the monoclonal antibodies that have entered the trial space are summarized in Table 1. 

Recent efforts have also focused on developing novel antibodies against various components of the FGFR2b signaling cascade in an effort to mitigate adverse effects seen in other monoclonals while maintaining therapeutic efficacy. For example, bemarituzumab has been shown to have significant corneal toxicity that appears to be associated with FGF10:FGFR2b targeting. Accordingly, GB2102 is a monoclonal antibody with potent FGF7 blocking activity, a known activating ligand of FGFR2b, but weak inhibition of FGF10. Early preclinical results have shown promising anti-tumor activity and a favorable safety profile, suggesting potential for clinical development [47].

### 3.3. FGFR2b Targeting with Immunotherapy

With the advent of immunotherapy now being the standard of care for some patients with metastatic gastric malignancies, the efficacy of FGFr2B-directed therapy alone or in combination with immunotherapy is now a clinical necessity. Interestingly, initial correlative studies have revealed that approximately 60% of FGFR2b-positive gastric cancer populations are also PD-L1 positive [48]. The combination treatment of FGFR2b and immunotherapy was studied in preclinical studies, which showed increased efficacy with the combination of bemarituzumab and anti-PD1 in an orthotopic mouse model (Figure 2D) [49]. With these preclinical and correlative data, ongoing trials like FORTITUDE 102, summarized in Table 1, investigate the combination of bemarituzumab and nivolumab.

**Table 1:** Ongoing trials of FGFR2 targeting in gastric cancer *.

Number	Trial ID	Phase	Eligibility	Design	Target	Enrollment	Primary Endpoint	Status	Estimated Completion	Name of Drug	Intervention
1	NCT05052801	Phase 3	Gastric Cancer, Gastroesophageal Junction Adenocarcinoma	Randomized, Double-Blind, Parallel Assignment	FGFR2b Overexpression	516	Overall Survival, Progression-Free Survival	Recruiting	18 August 2025	Bemarituzumab	mFOLFOX6, Placebo
2	NCT05859477	Phase 2	Metastatic Gastric Cancer, PD-L1 Gene Amplification	Single Group, Open Label	FGFR2 Amplification	23	1-Year Progression-Free Survival	Recruiting	December 2025	Nivolumab	Capecitabine, Oxaliplatin
3	NCT05019794	Phase 2	Gastric Cancer, Gastroesophageal Junction Adenocarcinoma with FGFR2 Amplification	Parallel Assignment, Open Label	FGFR Alterations	80	Objective Response Rate	Recruiting	30 December 2023	Infigratinib	N/A
4	NCT05111626	Phase 3	Gastric Cancer, Gastroesophageal Junction Adenocarcinoma	Randomized, Double-Blind, Sequential Assignment	FGFR2b Overexpressed	528	Part 1: DLTs, TEAEs	Recruiting	26 September 2026	Bemarituzumab, Nivolumab	Chemotherapy, Placebo
5	NCT01795768	Phase 2	Gastric Cancer, Oesophageal Cancer, Breast Cancer, Squamous Cell Carcinoma of the Lung	Single Group, Open Label	FGFR1- or FGFR2-Amplified Tumours	48	Anti-tumor Activity, Safety	Unknown	September 2015	AZD 4547	N/A
6	NCT05945823	Phase 2	Siewert Type 1 GEJ Cancer	Randomized, Double-blind	FGFR2	500	Progression-free survival	Active, not recruiting	June 2025	XYZ-789	Futibatinib, Pembrolizumab Cisplatin 5-FU Oxaliplatin Leucovorin Levoleucovorin Irinotecan

* This information is available at https://clinicaltrials.gov/ (accessed on 16 March 2024).

### 3.4. Role of Liquid Biopsy

Liquid biopsy, involving the analysis of circulating tumor DNA (ctDNA) and other biomarkers from blood samples, offers a non-invasive method to monitor FGFR2 alterations and treatment response in gastric cancer. Liquid biopsy can provide real-time insights into tumor dynamics, detect emerging resistance mutations, and guide personalized treatment strategies, thereby enhancing patient management [50,51].

## 4. Future Perspective

FGFR2 is positioned as a promising prognostic marker and therapeutic target in gastric cancer; the activation of pathways such as MAPK, PI3K/AKT, and YAP1 by FGFR2 emphasizes FGFR2’s potential as a key target for therapy [40]. The predictive value of FGFR2 could help categorize gastric cancer patients who might gain the most from FGFR2-focused treatments, thereby advancing personalized medicine efforts. A better understanding of the network of FGFR2’s effector pathways will be critical in predicting and circumventing resistance mechanisms and enhancing therapeutic outcomes. Research into targeted FGFR2 inhibitors, both as monotherapies and in combination with chemotherapy or immunotherapy, has revealed promising anti-cancer effects in early studies.

However, several hurdles remain before the wide clinical adoption of FGFR2-focused treatments. Gastric cancer is a genetically and cellularly heterogeneous disease that inherently challenges the effectiveness of targeted therapies like FGFR2 blockers. The lack of reliable markers for forecasting reactions to FGFR2-centric treatments underscores the necessity for ongoing studies into marker discovery and validation. Detailed genetic analysis is vital for pinpointing patients most likely to respond to FGFR2-centered therapies.

Furthermore, the creation of FGFR2 inhibitors necessitates a delicate balance between effectiveness and tolerability, as evidenced by known toxicities of drugs like bemarituzumab. Future investigations toward understanding and circumventing resistance mechanisms will be critical in enhancing therapeutic outcomes. Combination therapies involving FGFR2 inhibitors and other treatment modalities, such as immunotherapy and chemotherapy, are promising approaches to overcoming resistance and improving efficacy.

Recent efforts in liquid biopsy technology offer a non-invasive method to monitor FGFR2 alterations and treatment responses in real-time, allowing for more precise and adaptive treatment strategies [50]. Integrating liquid biopsy into clinical practice could significantly improve patient management by providing timely insights into tumor dynamics and emerging resistance mutations [51].

Moreover, ongoing clinical trials are investigating various FGFR2-targeted therapies, both as monotherapies and in combination with other treatments. For instance, combining FGFR2 inhibitors with immune checkpoint inhibitors shows the potential to enhance anti-tumor efficacy through synergistic effects [49]. These trials will provide critical data on the safety and efficacy of these novel therapeutic strategies and help refine treatment protocols.

In summary, while FGFR2 represents a compelling target in gastric cancer therapy, the path to its effective clinical application is complex and requires continued research. By addressing the challenges of genetic heterogeneity, resistance mechanisms, and treatment tolerability, we can move closer to integrating FGFR2-targeted therapies into the standard care for gastric cancer. This will ultimately lead to more personalized and effective treatment options for patients battling this malignancy.

## 5. Conclusions

The exploration of FGFR2’s role in gastric cancer has unveiled its critical contribution to the disease’s pathogenesis and progression, particularly through its involvement in cell proliferation, survival, angiogenesis, and resistance to chemotherapy. The distinct molecular characteristics of FGFR2, including gene amplification and overexpression, have highlighted it as a promising target for novel therapeutic strategies aimed at improving patient outcomes. The advent of targeted therapies such as FGFR inhibitors and anti-FGFR2b monoclonal antibodies underscores a pivotal shift towards precision medicine in treating gastric cancer. However, the clinical application of FGFR2-targeted treatments faces challenges as outlined previously. The ongoing clinical trials and research into combination therapies will provide greater insight into the disease while hopefully providing clinically meaningful therapeutic benefits. As we advance, the dynamic landscape of gastric cancer treatment demands continued investigation into FGFR2’s molecular mechanisms and therapeutic potential, promising a future where more personalized and effective treatment options are available for patients battling this malignancy.
